# Hybrid histidine kinases and antifungal warfare in thermal dimorphic fungi

**DOI:** 10.1128/aac.01225-25

**Published:** 2026-02-04

**Authors:** Frances S. Faguy, Maciej Walczak, Bridget M. Barker

**Affiliations:** 1 Pathogen and Microbiome Institute, Northern Arizona University 825831, Flagstaff, Arizona, USA; 2 Department of Chemistry, University of Colorado 196215, Boulder, Colorado, USA; Samsung Medical Center Department of Infectious Diseases, Seoul, Republic of Korea

**Keywords:** Coccidioidomycosis, *Coccidioides posadasii*, *Coccidioides immitis*, fluconazole, ambruticin, hybrid histidine kinase

## Abstract

Thermal dimorphic fungal pathogens are fungi that infect humans, often through the inhalation of asexual conidia, and which transition from hyphae to yeast in the human body. These fungi cause severe or chronic mycoses and are typically treated with azoles or amphotericin B. Ambruticin, a polyketide antifungal, shows promise as an alternative therapy. It targets hybrid histidine kinases (HHKs), which are fungal-specific proteins essential for osmoregulation and parasitic morphology and are conserved across thermal dimorphic species. Targeting HHKs suggests that ambruticin may therapeutically treat infections from multiple fungi without causing mechanism-based toxicity. We explore ambruticin’s potential to effectively treat these fungal infections without major adverse effects.

## INTRODUCTION

Fungi have evolved to feed on diverse substrates and to present an array of morphologies, from multicellular hyphal bodies to unicellular yeasts (1). The thermal dimorphic fungal pathogens are a unique group of fungi that have evolved to exist in the environment as filamentous molds before colonizing human lungs as pathogens and forming yeasts or yeast-like structures (1). For thermal dimorphs, the increased temperature in mammalian lungs is one trigger that initiates the morphological transition from mold to yeast. This mold-to-yeast transition is essential for survival and propagation in the lungs (2). Thus, proteins responsible for the mold-to-yeast transition, including cell wall proteins and those necessary for cell wall restructuring, are important virulence factors and hold potential for antifungal drug development.

**Fig 1:**
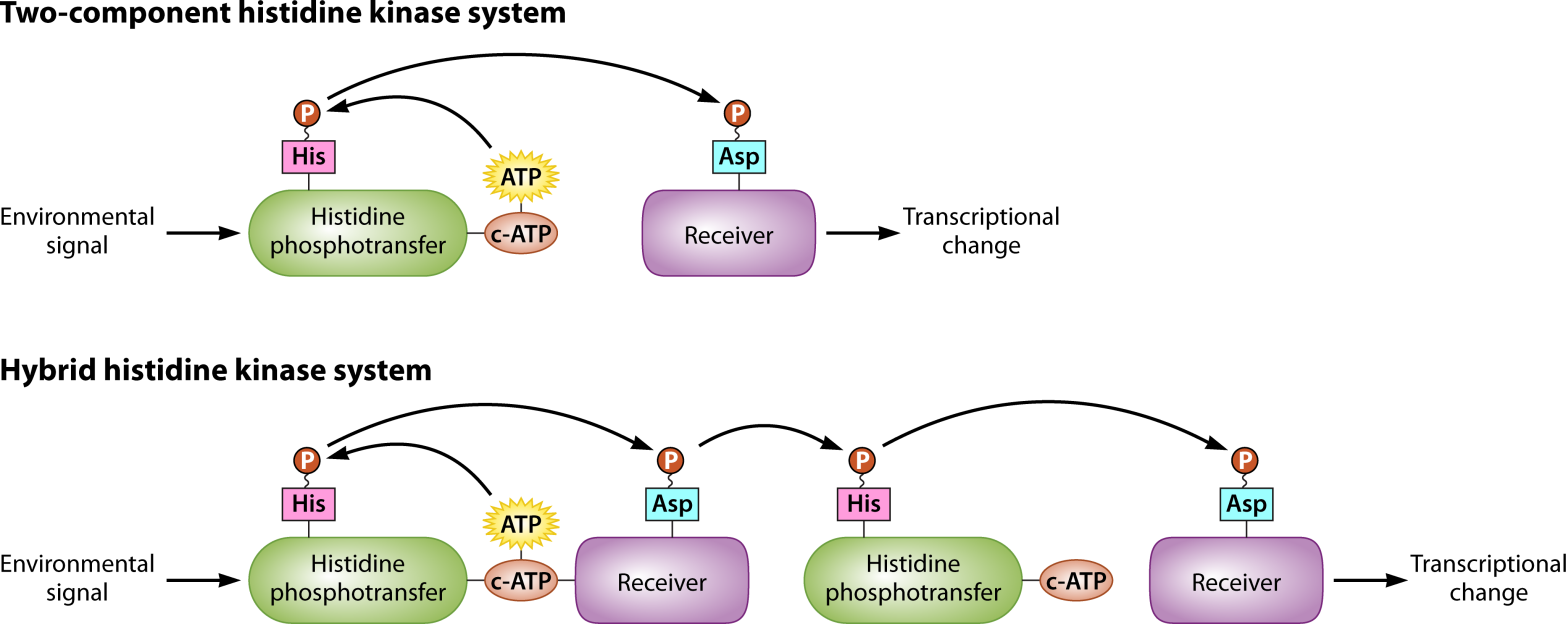
HHK system in fungi compared to a two-component histidine kinase system. The process of signal transduction within a two-component histidine kinase system is displayed, in which a phosphoryl group is transferred from a histidine residue on the histidine phosphotransfer domain to an aspartate residue on the receiver domain. In the HHK system, however, the phosphoryl group is then transferred from the aspartate residue to a second histidine residue on the histidine phosphotransferase domain, before it is transferred for a third time to a second aspartate residue on a second response regulator (15).

Hybrid histidine kinases (HHKs) are a class of signaling proteins found in all thermal dimorphs studied to date and are known virulence factors (Fig. 1). Class III HHKs are relatively conserved among thermal dimorphs, with two homologs present in most species: Drk1 and Nik1. Drk1, or dimorphism-regulating kinase 1, formerly known as Hik-1, is an HHK present in *Coccidioides immitis*, *Coccidioides posadasii*, *Histoplasma capsulatum*, *Blastomyces dermatitidis*, *Paracoccidioides brasiliensis* (PbDrk1), *Sporothrix schenckii* (SsDrk1), and in *Talaromyces marneffei* (DrkA) (3–10). Drk1 is known to be essential for virulence in *H. capsulatum*, *B. dermatitidis*, *P. brasiliensis*, and *S. schenckii* (3, 8, 10). Drk1, a relatively conserved protein throughout thermal dimorphs and putative target of the antifungal ambruticin, is a promising antifungal treatment target (11, 12). Although less is known about Nik1 function in the thermal dimorphs, Nik1 is well-studied in *Candida albicans* and can impact the efficiency of yeast formation (13, 14). In *Candida*, Nik1p contains nine HAMP domains (domains found in *h*istidine kinases, *a*denylate cyclases, *m*ethyl accepting proteins, and *p*hosphatases) (13, 14). Most orthologs of Nik1 are ~1k base pairs in length; most Drk1 orthologs are ~4k base pairs (4, 7, 10). The following review discusses six major thermal dimorphic pathogens, highlighting the difficulty clinicians experience when treating them. The review then describes the HHKs and discusses ambruticin as a promising therapeutic.

## PART I: THERMAL DIMORPHIC FUNGI

**Fig 2:**
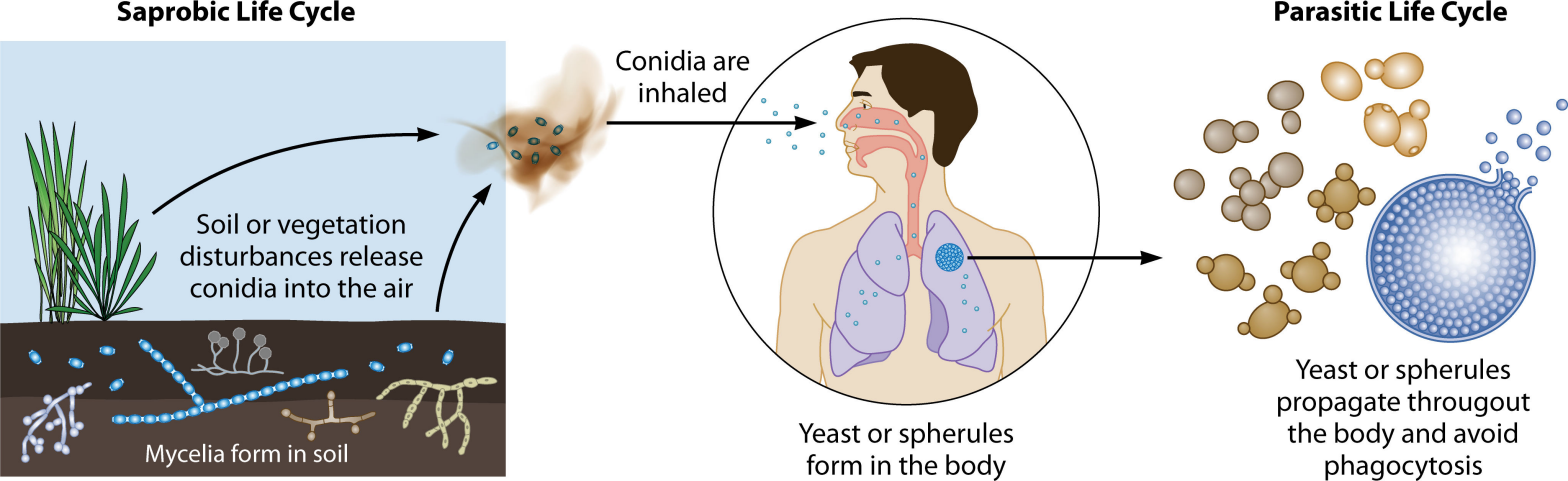
General thermal dimorphic life cycle. Infectious asexual particles called conidia are part of the environmental sparobic phase and variable yeast forms in the parasitic phase are depicted (4, 8, 9, 16–18). Although a human is pictured, thermal dimorphs can infect a variety of vertebrate hosts, especially burrowing or cave-dwelling animals. In some cases, the life cycle can continue after the host dies and the fungus re-enters the soil (19).

Thermal dimorphs are a group of pathogenic fungi that live as hyphae in soil but which complete a phase transition into yeasts or spherules in the host (Fig. 2) (2). The phase transition is often triggered by an increase in temperature from ambient (~25℃) to host body temperature (~37℃). The hyphal form is primarily the saprobic phase; the yeast phase is parasitic. Thermal dimorphic fungi cause millions of human infections per year, infecting both immunocompetent and immunocompromised people. There are six genera of thermal dimorphic human pathogens that are responsible for most of the clinical burden of disease and the focus of this review: *C. immitis* and *C. posadasii*, *P. brasiliensis*, *H. capsulatum*, *B. dermatitidis*, *T. marneffei*, and *S. schenckii*. For comparison and context, the pathogens *Candida spp.* and *Aspergillus spp.* are included (2).

Typically, thermal dimorphic fungal pathogens infect via inhalation, although sporotrichosis infections are most frequently acquired via the cutaneous route (20). Cutaneous coccidioidomycosis, blastomycosis, and talaromycosis are reported rarely (16–18). Although this review focuses on thermal dimorphs as human pathogens, thermal dimorphs commonly infect other mammals (18, 21). Most thermal dimorphs form yeast in mammalian lungs, but *Coccidioides* spp. form spherules, each of which can release hundreds of endospores upon maturation (19, 21). Until recently, the cellular and molecular biology of thermal dimorphic fungal pathogens has been poorly understood; however, CRISPR gene editing has been developed as a tool to investigate non-model fungal species, including the thermal dimorphic pathogens (22, 23).

Transcription of genes encoding virulence factors in thermal dimorphic pathogens is typically upregulated during the parasitic phase as opposed to the saprobic phase. Often, these virulence genes are involved in the phase transition itself, in which the cell is restructured. This requires coordination of chitin-building genes, chitinase genes, transcription factors, and signaling pathways which cause transcriptional changes based upon sensing changes in the environment (3). HHKs act as an essential step in the high-osmolarity glycerol (HOG) signaling pathway (discussed in further detail below) and are essential for the phase transition. In *H. capsulatum*, *B. dermatitidis*, *P. brasiliensis*, and *S. schenckii*, the loss of HHK function prevents the transition into the yeast phase (3, 8).

Other virulence factors conserved among thermally dimorphic fungi have roles in the morphological switch, although much of the evidence for virulence is based upon homology. Calcium signaling, a process involved in the thermal dimorphic phase transition, as well as the adaptation to pH and osmotic changes, is associated with virulence. In *H. capsulatum*, the calcium-binding protein Cbp1 is both yeast-phase-specific and essential for virulence (24). In *B. dermatitidis*, the TNF-α blocker BAD-1 is another yeast-phase-specific virulence factor (25). Heat-shock proteins, a class of proteins whose genes are significantly upregulated as a response to sudden heat stress in both prokaryotes and eukaryotes, allow thermal dimorphs to adapt to their host environment. In *P. brasiliensis*, several heat-shock proteins are expressed during the phase transition as well as later during the yeast phase (26–28). Ryp1 is a transcription factor in *H. capsulatum* and *Coccidioides* spp. and an ortholog of the Wor-1 protein in *C. albicans* and the Mit1 protein in *Saccharomyces cerevisiae* (29–32). Ryp1 is essential for both morphological changes and virulence in *Coccidioides* and in *H. capsulatum* (30–32). The correlation between signal transduction proteins and virulence indicates that the morphological switch is a strong target in drug development for dimorphic pathogens.

### *Coccidioides* spp.

*C. immitis* and *Coccidioides posadasii* are the only species in the *Coccidioides* genus and cause coccidioidomycosis, also known as Valley fever. *C. immitis* is endemic to central and Southern California, Western Mexico, and Eastern Washington. *C. posadasii* is endemic to arid regions in Arizona, Utah, Texas, Mexico, and Central and South America (33). Although *Coccidioides* is endemic to several arid regions in the Americas, 97% of infections between 2011 and 2017 were reported from California and Arizona (34). Coccidioidomycosis infects thousands of people annually in the reported endemic areas, with 20,061 confirmed cases reported in 2019 alone (35). According to a 1946 report by Smith et al. (36), 60% of *Coccidioides* infections are asymptomatic. However, the sample in the study was relatively small and homogenous, and a more recent estimation has not been reported. Symptomatic patients, which include both immunocompetent and immunocompromised people, experience a range of symptoms, most commonly acute or chronic pulmonary symptoms including coughing, chest pain, and, in some cases, erythema nodosum, along with fever and skin lesions (37, 38).

*Coccidioides* may also disseminate from the lungs; this is notably likely for those with AIDS or lymphoma, or those undergoing treatment with high-dose corticosteroids, TNF blockers, and JAK inhibitors (39, 40). *Coccidioides* may affect a wide range of tissues, most commonly the skeletal, lymphatic, and/or central nervous system (39, 41). Coccidioidal meningitis, defined as dissemination to and swelling of the meninges surrounding the brain, is lethal to those who do not receive antifungal treatment (42, 43). In addition to humans, their pets, and livestock, *Coccidioides* infects a variety of wildlife and is hypothesized to rely on desert rodents as preferred hosts in an endozoan life cycle (21).

Coccidioidomycosis is most often acquired by the inhalation of arthroconidia (44), but it can be acquired cutaneously (16). Rather than forming yeast in mammalian lungs, *Coccidioides* arthroconidia form thick-walled structures, called spherules, of up to 120 μm in diameter, which can erupt and release hundreds of endospores (diameter of 2–4 μm) although these size ranges have not been assessed in many strains (Fig. 2 [44]). Spherule development can be observed *in vitro* as well (45). Upon release, endospores can begin transforming into spherules and produce new endospores, thus continuing the parasitic life cycle (46). When in the human body, an endospore can be phagocytosed by a single neutrophil, but a spherule cannot be easily phagocytosed due to its large size and neutrophils instead exhibit frustrated phagocytosis (46). Thus, spherule development is thought to contribute to *Coccidioides* virulence. In mammalian hosts, upon host death, *Coccidioides* can revert to a hyphae and may re-enter the soil and feed off the decaying host (46). *Coccidioides* is a difficult pathogen to study, and there are limited published methodologies for its growth and transformation, even compared to other thermal dimorphic fungi (45).

In *Coccidioides*, multiple genes are differentially expressed between the saprobic phase and the parasitic phase, including the spherule outer wall glycoprotein (SOWgp), chitin synthetases, and chitinase genes (47). SOWgp acts as an adhesin in host lungs and is expressed during the parasitic life cycle. When SOWgp is deleted, *Coccidioides* demonstrates significantly lower mortality in a murine model than the wild-type strain (48). The *Coccidioides* HHK Drk1, homologous to Drk1 in *B. dermatitidis* and *H. capsulatum*, may have a similar contribution to virulence (3, 6, 9).

### *Paracoccidioides* spp.

*Paracoccidioides* is a thermal dimorphic fungal genus found in much of South and Central America and is the causative agent of paracoccidioidomycosis. The *Paracoccidioides* genus was expanded upon the discovery of new species *P. brasiliensis sensu stricto*, *Paracoccidioides americana*, *Paracoccidioides venezuelensis*, and *Paracoccidioides lutzii* (49). Paracoccidioidomycosis is acquired via the inhalation of conidia, which may infect animals as well as humans (49). In mammalian lungs, at 37℃, *Paracoccidioides* spp. form multibudding yeast cells, in which small buds extend from a large central cell (49, 50). *Paracoccidioides* frequently disseminates and can cause a range of symptoms, including symptoms in the lymphatic system, the central nervous system, the digestive system, and the upper aerodigestive tract, along with skin lesions (49). The most common pulmonary symptom of paracoccidioidomycosis is dyspnea, and pulmonary function is often impaired (49). Although paracoccidioidomycosis can present as an acute or subacute mycosis, particularly in adolescents, approximately 80% of cases are considered chronic infections (49).

Glycoprotein 43 (gp43) is a yeast-phase specific virulence factor in *P. brasiliensis*. A gp43 deletion mutant demonstrated significantly reduced virulence in a murine model (51). Other genes upregulated during the *P. brasiliensis* phase transition include the heat-shock proteins Hsp30, Hsp70, Hsp82, and Hsp104 (26–28). *P. brasiliensis* also has a Drk1 protein, PbDrk1, which contributes to yeast morphology and is necessary for virulence (7, 10). PbDrk1-silenced cells did not exhibit standard yeast morphology, instead resembling pseudohyphae, and the strains exhibited reduced virulence in a *Galleria mellonella* model (10).

### *Histoplasma* spp.

*H. capsulatum*, the main causative agent of histoplasmosis, is a thermal dimorph endemic to much of the Eastern United States and has a global distribution. Initially, its suspected endemic area was limited to regions surrounding the Ohio and Mississippi river valleys; however, it has been found in other humid regions in the United States with acidic soil and with high prevelence and diversity in South America (52–54). The species complex known to cause histoplasmosis has been revised. *H. capsulatum sensu stricto* has been joined by *Histoplasma ohiense*, *Histoplasma mississipiense*, and *Histoplasma suramericanum* in the Americas (54), and *H. capsulatum* var. *duboisii* in central Africa, although sampling across the continent is poor (55). Diversity and prevalence in Eurasia are beginning to be explored. Histoplasmosis is acquired by inhaling microconidia aerosolized from surrounding soil (56). Primary histoplasmosis presents with virus-like pulmonary symptoms, while chronic histoplasmosis can also cause bronchial damage and bullous emphysema (57). Histoplasmosis is among the most common pulmonary mycoses; in 2017 alone, global histoplasmosis infection totaled an estimated 500,000 cases, and global disseminated histoplasmosis was estimated at 100,000 cases (58).

Several *H. capsulatum* virulence factors have demonstrated increased expression during the yeast phase as compared to the mycelial phase. Calcium-binding protein 1 (CBP-1) is a virulence factor that allows *H. capsulatum* to proliferate in the limited-calcium environments of mammalian lungs (27, 59). As with other virulence factors, yeast-phase *H. capsulatum* cultures produce CBP-1, while mycelial *H. capsulatum* cultures do not (59). Deletion of a yeast-phase-specific cell wall polysaccharide necessary for the formation of the yeast cell wall, 1-3-α-glucan, also prevents the phase transition and severely attenuates virulence in a murine model (60, 61). Another contributor to *H. capsulatum* virulence is the aptly named yeast-phase specific protein 3, which is found on the cell wall surface and may also be ejected from the cell wall (62, 63). *H. capsulatum* has a gene homologous to the *Candida* Wor1 gene that binds upstream of various transcription factors and genes, which causes developmental changes crucial for *Candida* virulence. The homolog of Wor1, Ryp1, is essential for *H. capsulatum* virulence as well as for the phase transition (30). As with previous examples, Drk1 is necessary for the phase transition in *H. capsulatum* (3). Drk1 in *H. capsulatum* was knocked down via RNA interference (RNAi), and the resulting strain did not fully transition into the yeast phase at 37℃, instead forming pseudohyphae (3). The Drk1 deletion strains also demonstrated reduced virulence in a murine model upon intratracheal inoculation (3). Interestingly, the effects of Drk1 deletion are enhanced when expression of virulence factors Cbp1 and 1,3-ɑ-glucan is reduced (3). It is possible that Drk1 and the virulence factors Cbp1 and 1,3-ɑ-glucan impact similar transcription factors.

### *Blastomyces* spp.

*B. dermatitidis* is the primary causative agent of blastomycosis, a relatively rare but serious mycosis, and much less is known about its genetic diversity and global distribution. Blastomycosis is acquired via the inhalation of aerosolized conidia and most commonly presents pulmonary symptoms (64). However, inhalation-acquired blastomycosis can progress to cause skin lesions, osteomyelitis in the bones, and genitourinary and central nervous system symptoms (65). Blastomycosis affects both immunocompromised and immunocompetent individuals but presents a disproportionate risk to the immunocompromised. The total case fatality rate is estimated between 4% and 22%; however, for immunocompromised patients, a staggering 25% to 54% of blastomycosis cases are fatal (66). *B. dermatitidis* is highly endemic to regions surrounding the Great Lakes in central North America. However, blastomycosis cases have been reported throughout North America, as well as in India, Africa, and areas of Europe, and may be caused by different species (65).

Several virulence factors have also been identified as yeast-phase specific. Bad1 is an abundant *B. dermatitidis* surface protein, deletion of which severely attenuates virulence (67, 68). Bad1 imparts virulence in two ways; it allows *B. dermatitidis* yeast to adhere to pulmonary cells, and it prevents TNF-α production by host macrophages, inhibiting the host’s innate immune response (68). Just as in *H. capsulatum*, the histidine kinase Drk1 is a virulence factor in *B. dermatitidis* and is essential for its phase transition, and loss of function prevents a complete transition out of the mold phase (3). Drk1 was silenced in *B. dermatitidis* via RNAi, and the resulting strain exhibited pseudohyphal morphology rather than yeast morphology at 37℃ (3). The Drk1-silenced strains also had severely attenuated virulence in mice after intratracheal inoculation. Additional effects of Drk1 silencing were observed as Drk1-silenced strains did not upregulate expression of Cbp1 and Bad1 to wild type levels upon a temperature increase to 37℃ (25).

### *Sporothrix* spp.

The *Sporothrix* genus represents a unique set of thermal dimorphic fungal pathogens, which are typically transmitted through the skin rather than through the respiratory system (20). Sporotrichosis, the *Sporothrix*-associated mycosis, is most often caused by *Sporothrix brasiliensis*, *S. schenckii*, or *Sporothrix globosa*, although the species complex is still being described. Historically, sporotrichosis was most often contracted from the environment, particularly after contact with soil, plants, or decaying wood (20, 69). However, in recent decades, *S. brasiliensis* has caused a dramatic rise in zoonotic sporotrichosis, primarily transmitted from felines (69). *S. brasiliensis* is mostly acquired through traumatic inoculation routes, such as cat scratches or bites. It may also be acquired via non-traumatic inoculation, through contact with skin lesions on ill cats or via droplet exposure from cat sneezes (69, 70). Unlike other thermal dimorphic mycoses, sporotrichosis most commonly manifests as a subcutaneous infection, forming lesions at the site of infection (20). Sporotrichosis may also affect the mucous membranes, joints, bones, and lungs (71), as well as the eyes (20). *Sporothrix spp.* are significantly more resistant to antifungals, with both AmB and ambruticin S having higher *in vivo* minimum inhibitory concentrations (MICs) against *Sporothri*x than against other thermal dimorphic fungi (72). *Sporothrix* species have a global distribution; sporotrichosis cases have been reported in Africa, Australia, Asia, and the Americas (73).

A diverse set of proteins is associated with *Sporothrix* virulence. These include the glycoprotein gp70, which acts as an adhesin in host lungs (74). Additionally, the heat-shock protein Hsp60 and the protein Pap1, both bound to the *Sporothrix* yeast cell wall, demonstrated significant contributions to virulence in a *G. mellonella* model (75). Similarly, the heat-shock protein HSP90 and the calcium/calmodulin kinase Sscmk1 are both essential for yeast formation (76). Like *H. capsulatum*, *P. brasiliensis*, and *Coccidioides* spp., *S. schenckii* has a Drk1 histidine kinase, called Ssdrk1, which makes a significant contribution to *Sporothrix* virulence. SsDrk1 is significantly upregulated in the yeast phase as opposed to the mycelial phase (3, 5, 77). SsDrk1 deletion mutants of *S. schenckii* were slowed in their transition to the yeast phase and had moderate virulence in a murine model (8). *Sporothrix* spp. share HHK orthologs with their thermal dimorphic relatives. Interestingly, SsDrk1 in *S. schenckii* is homologous to Drk1 in *T. marneffei*, *Coccidioides*, *P. brasiliensis*, *B. dermatitidis*, and *H. capsulatum* and dissimilar to HHKs present in the non-thermal dimorph *C. albicans* (5). Similarities in Drk1 across the species suggest a universal role in thermal dimorphism, but this has yet to be shown.

### *Talaromyces* spp.

Talaromycosis is caused primarily by the thermal dimorphic pathogen *T. marneffei*, formerly known as *Penicillium marneffei* (78). Since tracking began in the 1990s, *T. marneffei* has caused a reported 288,000 infections throughout tropical and subtropical Southeast Asia as of mid-2022 (79). Recently, two novel *Talaromyces* species (*Talaromyces phuphaphetensis* and *Talaromyces satunensis*) were isolated from soil inside a cave in Thailand, suggesting that work remains in characterizing the genetic structure of this genus (80). *Talaromyces* spp. are thought to infect via inhalation, ingestion, and skin inoculation (18). Talaromycosis has the highest burden of disease among HIV-positive individuals and is often fatal in those with HIV who do not receive treatment (81). Clinical manifestations of talaromycosis differ between immunocompetent and immunocompromised individuals, but most patients experience anemia, fever, and weight loss (18). *T. marneffei* has a Drk1 homolog (DrkA) which influences the phase transition and is critical for pathogenesis, just as Drk1 does for other thermal dimorphs. It also has another histidine kinase, a Sln1 homolog (SlnA) which has a similar role to Drk1, just as Sln1 does in other thermal dimorphic pathogens (4).

### *Aspergillus* spp.

*Aspergillus* species, primarily *Aspergillus fumigatus*, are responsible for aspergillosis, the most common pulmonary fungal infection, which also presents a diverse range of symptoms in different patient populations. In patients with asthma or cystic fibrosis, *Aspergillus* can cause allergic bronchopulmonary aspergillosis. In relatively immunocompetent patients with underlying lung disease, *Aspergillus* can cause aspergillomas, nodules, and fibrosis characteristic of chronic pulmonary aspergillosis; in immunocompromised patients, *Aspergillus* can cause nonspecific symptoms of invasive pulmonary aspergillosis (IPA) (82). Like the thermal dimorphs, *Aspergillus* conidia enter the respiratory system, but upon colonization of the lungs, *Aspergillus* conidia emerge as hyphae rather than yeast. *Aspergillus* is present in remarkably diverse soil conditions which vary in temperature, pH, humidity, moisture, and oxygen concentration, but it is most common in tropical and subtropical regions (83). *A. fumigatus* has a group III HHK, NikA, a homolog of Nik1 in thermal dimorphs, which acts as part of the HOG pathway and is necessary for conidiation and for osmotic stress responses (11). It also has the HHK Fos1, deletion of which attenuates virulence (84, 85). Despite having characterized HHKs, *Aspergillus* does not appear to have a Drk1 ortholog, unlike the thermal dimorphic fungi.

### *Candida* spp.

*Candida* spp. are the causative agents of candidiasis, and their HHKs are known virulence factors. Unlike dimorphic fungi, which switch between two morphologies, *Candida* is technically a polymorphic fungus, switching between hyphal, pseudohyphal, and yeast morphologies (13). In addition to the temperature changes that cause thermal dimorphs to transition, *Candida*’s transition is influenced by pH and nutrient availability changes (86). *Candida* is generally excluded from the list of thermal dimorphic pathogens, although its virulence-associated dimorphism has been well-characterized (14, 87, 88). Unlike thermal dimorphic fungi, *Candida* does not commonly infect via the respiratory system; rather, it is associated with gastrointestinal tract infections, bloodstream infections, and vulvovaginal candidiasis (86). Candidiasis occurs globally and is caused by an extensive list of species, including *Candida glabrata*, *Candida parapsilosis*, and *Candida tropicalis* (89), many of which have undergone phylogenetic naming changes. In addition, the multidrug-resistant species *Candida auris* has brought global attention to fungal diseases (89, 90).

Although *Candida* spp. are not generally classified as thermal dimorphic pathogens, they switch from a hyphal morphology to a yeast morphology when grown at 37℃ *in vitro* (8). *Candida* species also have HHKs which mirror the function of those in thermal dimorphic pathogens. *Candida* has a Nik-1 homolog, Ca-Nik1, with high sequence similarity to Nik-1 in *Neurospora crassa* (91, 92). Deletion of Ca-Nik-1 in *C. albicans* also reduced the efficacy of its morphological switch, demonstrating a similar function to that of HHKs in thermal dimorphic pathogens (92). Additionally, the HHK CaSln1, a homolog of the *S. cerevisiae* Sln1, demonstrated a similar phenotype. Deletion of CaSln1 and CaNik from *C. albicans* prevented yeast formation (92). Thus, HHKs in *Candida* share some structure and function with those in thermal dimorphs. However, like the other non-thermal dimorph *Aspergillus*, *Candida* does not have a Drk1 ortholog annotated.

## PART II: HHKS AND THE HOG PATHWAY

**Fig 3:**
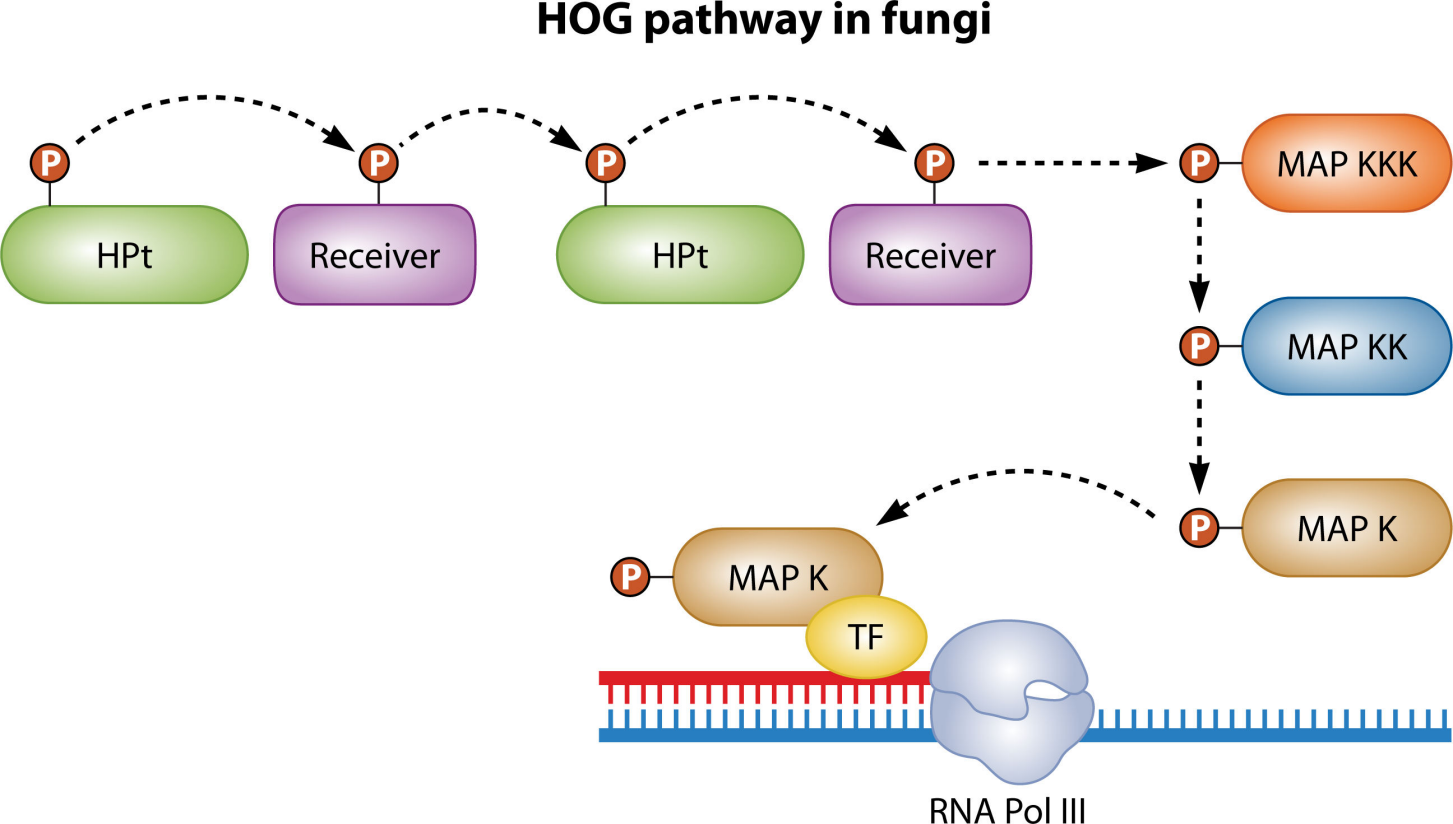
HOG pathway in fungi. Environmental signals are relayed through the HOG pathway in fungi via the HOG pathway. First, a HHK completes three phospho-transfers as demonstrated in Fig. 1. Next, a series of MAPKs relay the phosphoryl group. First, a MAPKKK receives the phosphoryl; then, a MAPKK, and finally a MAPK receives the group. The MAPK activates a transcription factor and initiates transcription of the appropriate gene.

HHKs are signal relay proteins that allow fungi to adapt to environmental changes (Fig. 3). Specifically, HHKs are thought to trigger the phase transition in thermal dimorphic fungal pathogens. HHKs are unique to fungi; other eukaryotes and many prokaryotes have two-component histidine kinases instead (3). In these two-component systems, a histidine kinase sensor domain, also called a histidine phosphotransfer domain, autophosphorylates before transferring its phosphoryl group to a response regulator protein, which initiates transcription of appropriate genes (15, 93). However, the HHKs are part of a three-protein histidine kinase system that transfers a phosphoryl group to three residues sequentially. The HHK contains a histidine phosphotransfer domain that autophosphorylates before transferring its phosphoryl group to a receiver domain. Then, the histidine kinase phosphorylates a histidine phosphotransferase protein, which in turn phosphorylates a response regulator (15, 94). The histidine phosphotransfer domain and the receiver domain form a single protein, of which some fungi have multiple and some only have one (94). The histidine phosphotransferase and response regulator domains comprise a second protein (94).

Fungal HHKs have been sorted into 16 groups based on their N-terminal sequence and the N-terminal sequence is conserved within each group but variable between the 16 groups (15, 95). Drk1 and Nik1 are both group III HHKs, meaning that they each have multiple repeats of the HAMP domain (96). Group III HHKs are the most conserved fungal histidine kinase class and are also the most common, making the class a promising antifungal target (97).

HHKs act as part of mitogen-activated protein kinase (MAPK) pathways, which act as signaling cascades, mediating gene expression as a response to environmental stimuli. In a MAPK cascade, a HHK senses an environmental change and begins a phospho-relay cascade, phosphorylating a series of kinase proteins. This phospho-relay cascade continues from a MAPKKK to a MAPKK to a MAPK. The HOG pathway is a classic example of a MAPK pathway, sensing increases in osmolarity and activating transcription of genes necessary for glycerol accumulation, thus balancing osmolarity inside and outside the cell (98, 99).

The HHK Drk1 is of particular interest in thermal dimorphs, as it may regulate thermal dimorphism itself. Drk1 is an HHK known to be essential for virulence in *H. capsulatum*, *B. dermatitidis*, *P. brasiliensis*, and *S. schenckii* (3, 5, 8, 10) and present in *C. immitis*, *C. posadasii*, and *P. brasiliensis* (6, 7). Drk1 in *H. capsulatum* and *B. dermatitidis*, along with SsDrk1 in *S. schenckii*, is necessary for the phase transition from hyphae to yeast (3, 5, 8). Based upon the genetic and phenotypic similarity across Drk1 homologs among the thermal dimorphs, it is likely that Drk1’s impact on virulence and its role in the phase transition is conserved. Thus, Drk1 is a likely key regulatory protein among thermal dimorphs.

## PART III: THE SEARCH FOR ANTIFUNGAL THERAPIES

Current treatment regimens for thermal dimorphic mycoses commonly employ azoles, either in place of or in combination with amphotericin B (AmB). For talaromycosis, AmB is often a first-line therapy, and azoles are administered afterward (79). AmB, once considered the gold standard of antifungal treatment, is a large lactone ring and a product of fermentation from certain myxobacteria (100). Its efficacy as a broad-spectrum antifungal was first demonstrated in 1955 (101), and its structure was determined in 1970 (102). AmB binds directly to ergosterol, an essential component in fungal cell membranes, creating pores that cause leakage and eventual fungal cell death (100). Azoles are fungistatic agents that, rather than binding directly to ergosterol, interfere with ergosterol biosynthesis, and prevent growth (100, 103). There are five common azoles clinicians may prescribe for combating endemic pathogens: fluconazole, itraconazole, voriconazole, posaconazole, and isavuconazole (64, 103).

Fluconazole is commonly prescribed at 400 mg/day for chronic or disseminated coccidioidomycosis or 800 to 1,200 mg/day for coccidioidal meningitis (103, 104). While the majority (67%) of chronic or disseminated coccidioidomycosis patients respond to fluconazole, 28% to 39% of chronic pulmonary infections and 50% of bone and joint infections are reactivated after responding to fluconazole treatment (104). Thus, for chronic pulmonary or disseminated coccidioidomycosis, fluconazole is typically prescribed for at least 1 year, and clinical guidelines state that fluconazole for meningeal coccidioidomycosis should be administered indefinitely (105–107). Itraconazole has become the treatment of choice for paracoccidioidomycosis, demonstrating efficacy at oral doses of 100 to 200 mg daily (49). Fluconazole is also extensively studied as a paracoccidioidomycosis treatment and may be prescribed orally or intravenously at 400 mg per day (49). For talaromycosis, itraconazole is also a common treatment, but reactivation of infection is common (79). These frequent mycosis reactivations may be attributed to the fungistatic, rather than fungicidal, effect of azoles. While fluconazole has been associated with hepatotoxicity at doses of 400 mg/day or higher, it has not been associated with nephrotoxicity, which is a serious concern for clinicians prescribing AmB (103).

Few fungi exhibit resistance to AmB; it can inhibit *Coccidioides*, *Paracoccidioides*, *Histoplasma*, *Blastomyces*, *Sporothrix*, *Aspergillus*, and *Talaromyces* species efficiently; however, it must be administered intravenously in a clinical setting (108). AmB binds directly to ergosterol in fungal cell membranes; however, it can also target cholesterol in mammalian cells, harming patients (109). AmB’s side effects have been attributed to this interaction with cholesterol (110, 111). The most serious adverse effect of AmB is nephrotoxicity, tissue damage to the kidneys (110). An estimated 12% of AmB patients experience nephrotoxicity, with one study in Palestine producing a much higher estimate of 50% (112, 113).

In addition to standard AmB formulations, a variety of liposomal formulations have been produced. These liposomes are of varying sizes and differ in both efficacy and toxicity between manufacturers (114). By far the best characterized among the available liposomal AmB formulations is AmBisome, which is 77% effective in treating invasive aspergillosis that had not responded to conventional AmB (115). AmBisome also resulted in significantly lower nephrotoxicity as well as lower overall toxicity than conventional AmB but still must be administered intravenously (114, 115). Thus, new antifungal therapies are needed. Given that the HHK pathway is highly conserved among several pathogenic fungi, this has been proposed as a druggable target.

**Fig 4:**
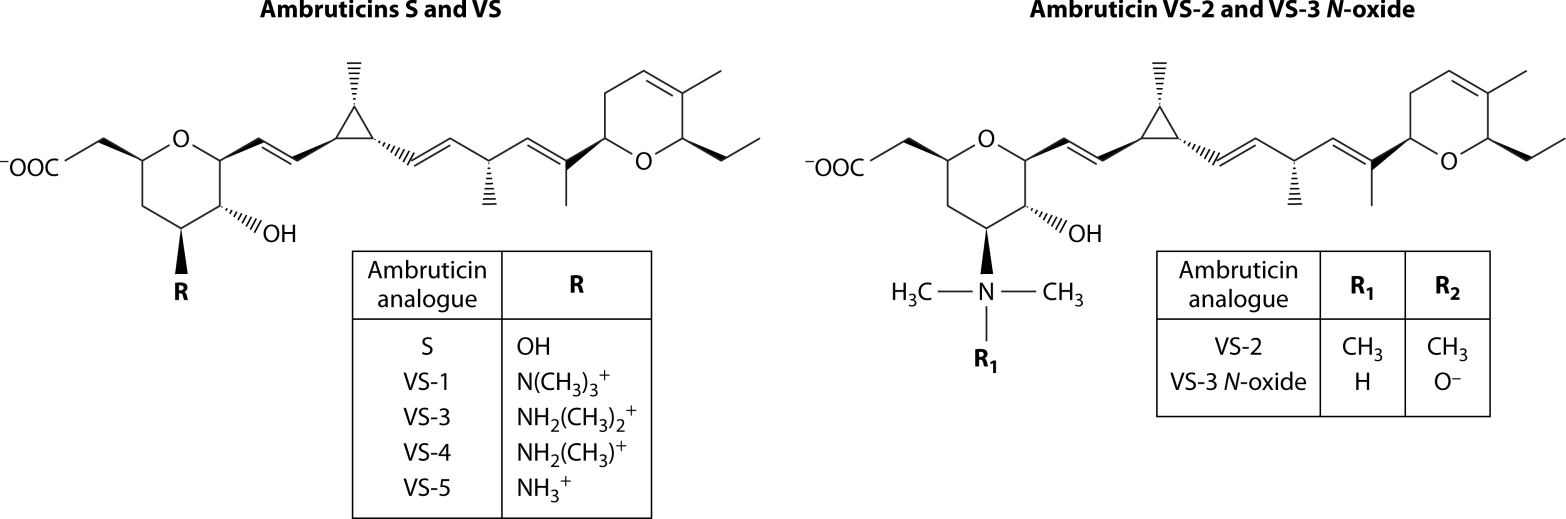
Ambruticin structure: ambruticin S, VS-1, VS -2, VS-3, VS -3 *N-*oxide, VS-4, and VS-5. Ambruticin with one R-group is shown in the left figure; ambruticin with two R-groups is shown on the right. Ambruticin S and ambruticin VS-1, VS -3, VS-4, and VS-5 have one R-group attached directly to their ring; ambruticin VS-2 and VS-3 *N*-oxide have an R-group attached to a nitrogen at the bottom of the ring. They have an additional R group attached to the carboxylate attached to the same ring. Structure derived from (117).

Ambruticin S is an antifungal polyketide produced by the myxobacterium *Sorangium cellulosum* (116). Multiple analogs of ambruticin S have been evaluated for efficacy, safety, and ease of synthesis. Ambruticin S was first discovered, and its structure first elucidated, by Ringel et al. in 1977. The paper presented a non-stereospecific structure which portrayed the epimers now known as ambruticin F and ambruticin S (116). In the same paper, several other structures were elucidated, which would later become ambruticin VS 1 through VS 5 and ambruticin VS-3-N-oxide (Fig. 4) (117). Analogs of ambruticin have also been produced and have demonstrated differing efficacy in treating murine coccidioidomycosis; analog KOSN-2089 demonstrated significantly improved survival and significantly reduced fungal burden compared to KOSN-2079 (108).

The *in vitro* MIC of ambruticin S have been described for multiple thermal dimorphs. Seven strains of *H. capsulatum* demonstrated *in vitro* MICs between 0.25 and 1 μg/mL (72). *C. immitis* demonstrated similar *in vitro* susceptibility to ambruticin S, with nine of 10 isolates tested having an MIC of ≤ 0.5 μg/mL (72). While *B. dermatitidis* demonstrated an MIC of 0.04 μg/mL, six strains later demonstrated MICs between 0.06 and 64 μg/mL (72, 117). In *S. shenckii*, 16 isolates demonstrated MICs between 4 and 16 μg/mL (72). In *P. brasiliensis*, however, the MIC was reported much higher, at 50 μg/mL (117).

Ambruticin S also demonstrated efficacy *in vivo* against *C. immitis* and *H. capsulatum*. Testing in a murine model was first performed with *C. immitis*, during which a treatment of 50 mg/kg of body weight per day reduced mortality from between 50% and 75% to zero percent (118). Ambruticin S demonstrated similar efficacy *in vivo* against *H. capsulatum* in a murine model, with a 50% cure dose between 75 and 150 mg/kg of body weight per day (119) (Table 1).

**TABLE 1:** Ambruticin and AmB MIC and therapeutic dosage in each thermal dimorph *in vivo* and *in vitro*

Thermal dimorph	AmB MIC (*in vitro*)	AmB dosage (*in vivo*)	Ambruticin S MIC (*in vitro*)	Ambruticin S dosage (*in vivo*)
*C. immitis*	0.5 μg/mL (71)	0.5–0.7 mg/kg/day (124)	0.25–4 μg/mL (71)	5–20 mg/kg/day (119)
*Paracoccidioides* spp.	0.25 μg/mL (130)	40 ug/day (murine) (130)	50 μg/mL (99)	
*Sporothrix* spp.	1–2 μg/mL (71)		4-128 μg/mL (*S. schenckii*) (71)	
*H. capsulatum*	0.06–0.25 μg/mL (71)		0.25–1 (71)	75–150 mg/kg/day (12)
*B. dermatitidis*	0.024–0.049 μg/mL (99)		0.04–64 (71)	

Ambruticin S *in vivo* dosage was later reported as 5–10 mg delivered either once or twice daily for coccidioidomycosis, which has been simplified to a maximum of 20 mg/kg/day in the above table (119). Future *in vitro* ambruticin S formulation efficacy can be evaluated using the Clinical and Laboratory Standards Institute M38 Reference Method for Broth Dilution Antifungal Susceptibility Testing of Filamentous Fungi.

**Fig 5:**
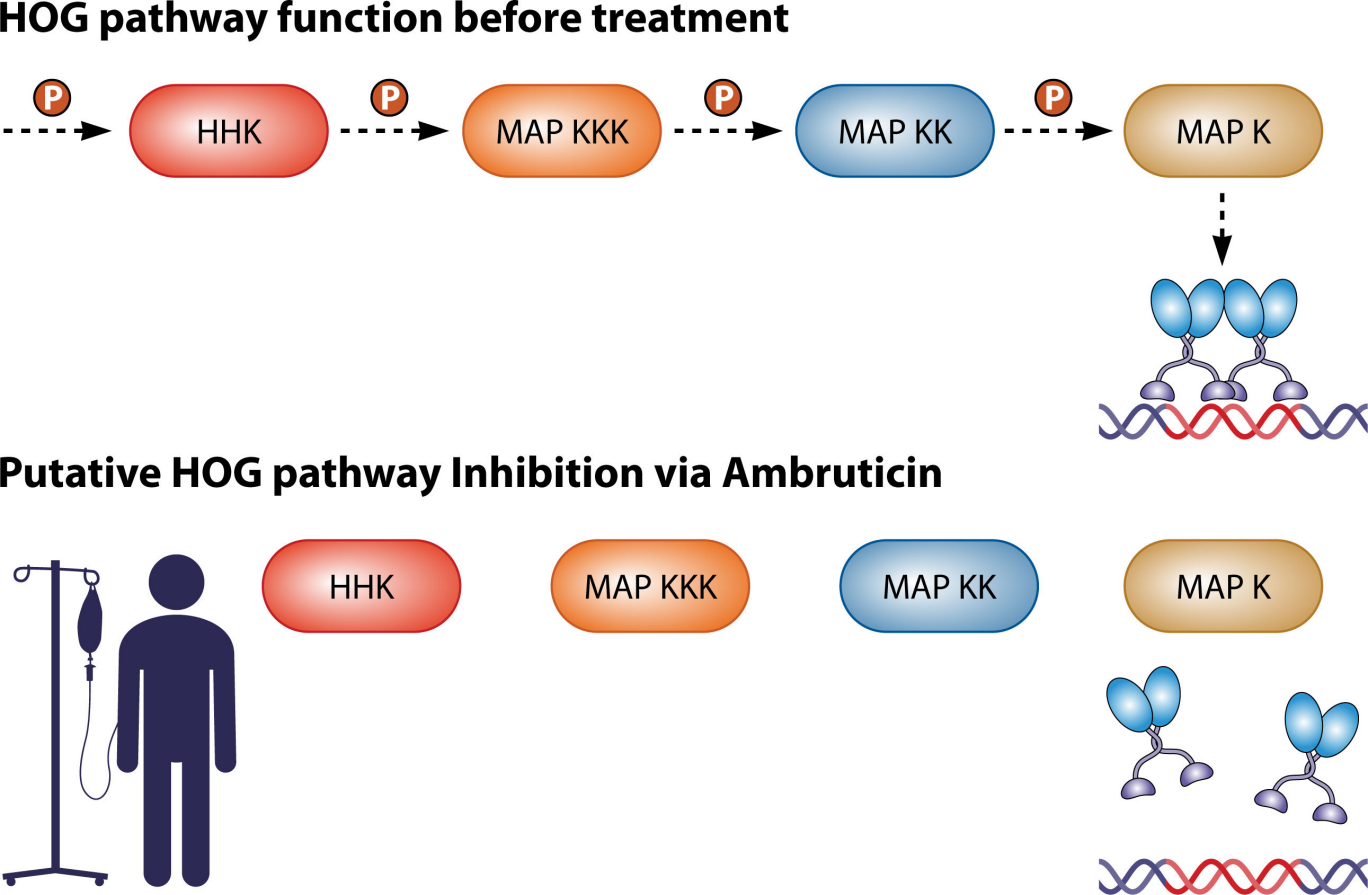
Putative ambruticin interactions with HOG pathway *in vivo*. The hypothesized ambruticin mechanism of action; ambruticin is thought to prevent phosphorylation of the HHK at the beginning of the HOG pathway, thus preventing the phosphoryl group from moving down the remainder of the HOG pathway. In turn, this prevents attachment of transcription factors to the promoters of morphological switch genes.

Ambruticin S disrupts osmoregulation, inducing glycerol accumulation in fungal cells (12). A Drk1 homolog from the rice blast fungus, *Magnaporthe oryzea*, confers ambruticin susceptibility to *S. cerevisiae*, which is not usually susceptible to ambruticin (120). The HHK Ca-Nik1, *Candida*’s Nik1 ortholog, also confers ambruticin susceptibility to *S. cerevisiae,* although Nik1 does not have a strong association with thermal dimorphism (121). Ambruticin activity against the plant pathogen *Alternaria brassicicola* is similarly dependent upon presence of the Nik1 homolog AbNik1 (121). It is hypothesized that ambruticin similarly targets the HHKs of the HOG pathway in thermal dimorphic fungal pathogens (Fig. 5). HHKs constitutively repress Gpd-1, which encodes a protein essential for glycerol production (122). While a functioning HHK allows for excess glycerol production only under osmotic stress, an HHK that has been deactivated by ambruticin is hypothesized to allow for Gpd-1 overexpression and subsequent glycerol production, which eventually leads to cell permeability and leakage that kills the fungus (108, 122).

## CONCLUSION

Thermal dimorphs are a group of fungal pathogens whose parasitic morphologies effectively colonize and damage host organs. Each thermal dimorphic pathogen has multiple HHKs, unique to fungi but relatively conserved between species. Notably, the HHK Drk1 is conserved among thermal dimorphs but apparently absent from their non-thermal dimorph relatives, *Candida* and *Aspergillus*. Drk1 is essential for virulence among thermal dimorphs, potentially making it an effective target for antifungal drugs. The antifungal drug ambruticin, along with its analogs, is effective against most thermal dimorphs *in vitro*. Ambruticin targets HHKs, indicating that it may be both effective and safe in treating thermal dimorphic mycoses. Given the difficulty in treating mycoses with other antifungals like azoles and AmB, ambruticin is a promising alternative treatment. Should ambruticin continue to show both efficacy and safety during *in vitro* testing, it may be moved to *in vivo* testing and eventually to clinical trials. Thus, ambruticin could eventually offer mycosis patients a more tolerable treatment option.
